# Episodic Future Thinking as an Intervention for Alcohol Use Disorder: Effects on Delay Discounting and Real-World Alcohol Consumption

**DOI:** 10.21203/rs.3.rs-9182511/v1

**Published:** 2026-04-03

**Authors:** Rafaela M. Fontes, Devin Tomlinson, Allison Tegge, Mikhail Koffarnus, Jeffrey Stein, Samuel McClure, Jeremy Myslowski, Rebecca Faubion-Trejo, James MacKillop, Anita Kablinger, Stephen LaConte, Warren Bickel

**Affiliations:** Fralin Biomedical Research Institute; McMaster University

## Abstract

Episodic future thinking (EFT) is a promising intervention for alcohol use disorder (AUD) as it has been shown to decrease delay discounting (DD) and alcohol valuation. In the current randomized controlled trial, we evaluated the effects of EFT on DD, behavioral economic demand, and daily measures of real-world alcohol consumption among individuals with AUD. Sixty-four non-treatment-seeking adults with AUD who wished to reduce or quit drinking but had no immediate treatment plans were randomized to EFT (n = 34) or control episodic thinking (CET; n = 30). Participants completed four in-laboratory sessions over the course of five weeks, and a follow-up in-laboratory session one month after the completion of the intervention. Additionally, alcohol consumption was monitored remotely for five weeks via a digital app. EFT exposure acutely decreased DD relative to baseline and CET. Additionally, daily exposure to the EFT, but not to CET, decreased the number of drinks/day and the number of drinks/drinking day. Significant changes in behavioral economic demand were not observed. Overall, the present findings suggest that EFT is a promising intervention to decrease alcohol use with effects on real-world alcohol consumption among individuals with alcohol use disorder, but alcohol demand is not a mechanism of this effect.

## Introduction

Alcohol use is a significant public health problem and contributes to more than 178,000 deaths annually in the US^1^. Nearly half of individuals aged 12 and older report past-month drinking, and 10% meet criteria for alcohol use disorder (AUD)^2^. Despite treatment availability, only 1 in 9 individuals with AUD benefit from medication^3,4^, and brief psychotherapeutic interventions yield only modest reductions in drinking^5,6^. Moreover, fewer than 8% of individuals with AUD receive any treatment^7^, highlighting the need for low-burden, scalable interventions that support behavior change and decrease daily alcohol consumption.

One promising approach is episodic future thinking (EFT), an intervention grounded in the science of prospection that prompts participants to think about positive future events that are likely to happen^8,9^. The application of EFT as an intervention for alcohol use is informed by the reinforcer pathology theory^10,11^, which posits that substance use is maintained by a combination of a preference for immediate over delayed outcomes and high substance valuation. According to reinforcer pathology, reinforcer value is determined by integrating expected benefits and harms over a temporal window. The temporal window can be quantified with delay discounting (DD), which refers to the relative preference for smaller sooner over larger later rewards^12^. Higher DD rates indicate shorter temporal windows and, thus, greater valuation of intense, reliable, and immediate reinforcers, such as alcohol, relative to reinforcers that accrue value over time, such as long-term health.

EFT has been shown to lengthen the temporal window (i.e., decrease DD) and thus reduce the relative value of immediate reinforcers in favor of delayed^8^. Notably, reinforcing value can be measured through hypothetical behavioral economic demand tasks^13–15^ and real-world consumption^16^. EFT has been shown to decrease both DD and valuation in alcohol^17,18^, cocaine^19^, and cigarette use^20–22^. However, examination of EFT effects on real-world alcohol consumption is limited. For example, Athamneh et al.^16^ showed decreases in DD and daily drinking following EFT in a sample of individuals with AUD, but alcohol consumption was monitored only for two weeks. To advance toward real-world implementation, more research is needed to evaluate the effects of EFT on alcohol use under naturalistic conditions. Therefore, the goal of the current randomized controlled trial was to expand on the previous findings and evaluate the effects of EFT on DD, behavioral economic demand, and real-world alcohol consumption over a longer time frame than has been done previously. We hypothesized that EFT would decrease these measures among non-treatment-seeking individuals with AUD.

## Materials and Methods

**Figure 1:**
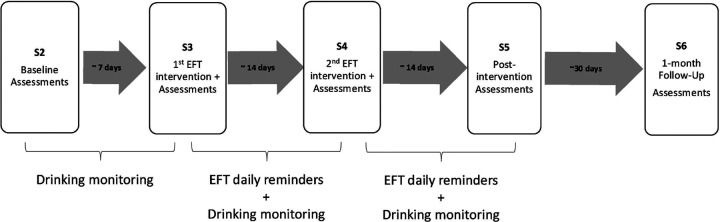
Study timeline.

This was a parallel trial that examined active (EFT) versus control conditions. Participation in the study lasted about nine weeks. Participants completed four in-laboratory sessions (S1–S4) and daily remote drinking monitoring over the course of five weeks, and a follow-up in-laboratory session (S5) one month after the completion of the intervention. All study sessions (S1–S5) were conducted in the laboratory and included a battery of behavioral assessments. During S2 and S3, participants also completed a resting-state MRI, and the neuroimaging results will be reported in a separate manuscript. Drinking monitoring was conducted remotely through a phone app. Figure 1 shows the study timeline. Total compensation was approximately $700 for completion of all sessions and remote submissions. Detailed compensation structure can be found in the Supplemental Materials. All procedures were registered at ClinicalTrials.gov (NCT04125238) and received ethical approval from the institutional review board at Virginia Polytechnic and State University (Protocol No. 22–358).

## Participants

Participants were recruited from the Roanoke, VA community via flyers posted in the community (e.g., bars, restaurants, grocery stores), word-of-mouth, electronic (e.g., Craigslist, Facebook, BuildClinical), and bus advertisements. All advertisement materials noted that this was not a treatment study, and participants were not informed about the aims of the study or the goal of the intervention. Eligibility was assessed with an online pre-screening questionnaire, including the Alcohol Use Disorder Identification Test (AUDIT), and DSM-5 questions about past 12-month substance use. Eligibility criteria to sign consent required participants to 1) be between 21–65 years old, 2) demonstrate high-risk or harmful drinking, defined as an AUDIT score ≥ 16, and 3) have a desire to quit or cut down their drinking, but no proximate plans to enroll in treatment. The goal of including individuals interested in reducing or stopping alcohol use was to focus on the relevant population for future studies. Participants were ineligible if they 1) met moderate to severe DSM-5 criteria for substance-use disorders other than alcohol, nicotine, or cannabis, 2) had a current diagnosis of a psychotic disorder, 3) had a history of seizure disorder or traumatic brain injury, 4) had contraindications for fMRI participation, or 5) reported current pregnancy or lactation.

Eligibility to continue in the study was assessed again after a seven-day baseline remote drinking monitoring phase (Baseline Monitoring), which occurred between S1 and S2 (Week 1). Only participants who reported their consumed number of daily drinks on at least five of the seven days and met criteria for harmful drinking were invited to continue in the study. Harmful drinking was defined as consuming alcohol on at least four of the seven days, with either 1) at least four days of consuming four or more drinks, and/or 2) an average of more than four drinks/drinking day. The purpose of the Baseline Monitoring phase was to quantify baseline drinking patterns and ensure that participants reliably responded to and conveyed study information remotely. Notably, there was only one exclusion exclusively due to a low number of submissions during baseline.

A power analysis assuming a conservative medium effect size (f = 0.25 based on previous studies^16,17^), a repeated measure correlation of 0.5, a Type I error rate of 0.01, and 80% statistical power indicated that 52 participants were needed to complete this study (n = 26 per group). G*Power was used to estimate sample size^23^. Because the longitudinal nature of the study and the baseline drinking requirements could contribute to high drop-out rates, we continued to enroll participants until 52 participants completed the one-month follow-up session (S5).

## Interventions

Participants who met the baseline drinking criteria were invited to continue in the study and randomized to EFT or control episodic thinking (CET). Participants were randomly assigned to EFT (n = 34) or CET (n = 30) with an even allocation ratio. As done previously^24–26^, the randomization procedure used a computerized algorithm that adjusted the probability of group assignment and biased the allocation to balance the groups on number of drinks/day (square-root transformed to reduce bias from a skewed distribution) and baseline DD rates. Participants in both groups underwent two interview-guided EFT/CET cue generation sessions at S2 and S3, and were exposed to their EFT/CET cues daily for four weeks (Weeks 2–5 between S2 and S4).

## Cue Generation

During the interview-guided EFT/CET cue generation sessions, participants in both groups were prompted to think vividly and describe in detail positive events for different time points. The only difference between the groups was the time orientation for the events. Those in the EFT group were prompted to think about events that would happen in the future (i.e., 1 day, 2 weeks, 1 month, 3 months, 1 year, 5 years, and 25 years in the future). Those in the CET group were prompted to think about events that occurred in the recent past (i.e., last night from 7 pm – 10 pm, yesterday between 4 pm – 7 pm, yesterday between 1 pm – 4 pm, yesterday from 10 am – 12 pm, yesterday between 7 am – 10 am, the night before last between 7 pm – 10 pm, and evening before last between 4 pm – 7 pm). Participants were prompted to think about one event per time point and asked a series of standard questions to help them think about the details of the event (e.g., where they are/were, who they are/were with, how they are/were feeling, etc.). Participants then provided a description of the event (long cue) and a short phrase to function as a reminder (short cue; see Supplemental Materials for examples of long and short cues).

## Cue exposure

Participants received reminders^16,27^ about their EFT/CET events (i.e., short cues) through SMS text twice daily (at 10am and 4pm). The short cues were texted in a random order (except for the 1-day EFT cue, which was not texted to the participants). The texts also included a randomly selected question designed to increase engagement with the cue (see Supplemental Materials for the complete list of questions), and participants received $1 for responding to it.

## Measurements

### Delay Discounting.

During each in-laboratory session (S1–S5), participants completed an adjusting-amount DD task, where they were presented with choices between smaller-sooner and larger-later amounts of money. The task included seven delays between 1 day and 25 years in the future, and six trials for each delay. The larger-later amount remained constant at $1000 across trials, and the smaller-sooner amount was titrated according to the participant’s previous choice, as described in Du et al.^28^. During S2 and S3, participants’ long cues were displayed above each choice (see Supplemental Materials for example). In the EFT group, cues were matched to their corresponding delay (i.e., 1-day cue shown during 1-day delay choices). In the CET group, the cues corresponded to their proximity to the present (e.g., last night from 7pm-10pm cue shown during 1-day delay choices). Cues were not shown during S1, S4, and S5.

### Alcohol purchase task (APT).

During each in-laboratory session (S1–S5), participants completed the APT where they were presented with a list of 13 prices in ascending order^29^ and instructed to indicate how many standard drinks (i.e., 12 oz beer, 5 oz glass of wine, 1.5 oz hard liquor) they would buy at each price. Prices ranged from $0.00 to $80/drink. Because APT does not include a temporal component, participants in both groups saw a list of their long cues on top of the price list in S2 and S3 (see Supplemental Materials for example). Cues were not shown during S1, S4, and S5.

### Alcohol Consumption.

During the first five weeks of the study (Weeks 1–5, between S1–S4), participants were required to report the number of drinks consumed per day and provide three breath samples/day. All participants were provided a BACtrack Mobile breathalyzer, an FDA-approved personal breathalyzer. Daily number of drinks and breath samples were measured using the Healthier Futures app developed for other ongoing research (R01AA026605) that connects to the BACTrack Mobile breathalyzer via Bluetooth. The app also collected an image of the participant’s face during breath submission for identity verification. The app was only available for Android, and participants who did not have an Android smartphone were provided with one (n = 39).

Every morning, participants were asked to report the number of alcoholic drinks they consumed the previous day via the app. Previous-day consumption, instead of same-day consumption, was preferred to best capture all drinks consumed each day without inconsistent response times compromising the accuracy of the measure. Participants received $1 for reporting their number of drinks, regardless of the results. Breathalyzer screens were spaced at random times throughout the day, spanning the participant’s waking hours. Participants had 90 minutes to complete each breath sample submission and received $1 per sample submitted within the time window, regardless of the result. To encourage participants to provide all daily submissions, participants received a bonus for each day in which all submissions were completed (see Supplemental Materials for payment structure for the remote monitoring). The remote monitoring lasted approximately 35 days (see Fig. 1). Because the length of the monitoring phases was contingent on session completion (i.e., baseline monitoring ended at S2, monitoring phase 1 ended at S3, and monitoring phase 2 ended at S4), the duration of the phases varied across participants (e.g., due to the participant or staff availability for the sessions). **Tables S1** and **S2** show mean and median length of each monitoring phase per group.

## Data Analysis

The Statistical Analysis Plan was pre-registered on the Open Science Framework (OSF) and can be found at https://osf.io/7vd6x/? view_only=5d2b2b498de9407fa1ed32251fbb1aa1. Any deviations from the SAP are noted in this section. All analyses were conducted using R software (version 4.2.2). Complete case and intention-to-treat analyses were performed for all outcomes. For the complete case analyses, only participants who completed all sessions, including the one-month follow-up (i.e., S5; EFT = 28, CET = 24) were included. For the intention-to-treat analyses, all participants who initiated the intervention (i.e., S2; EFT = 33, CET = 28) were included, and all sessions following discontinuation were treated as missing data. Additionally, for participants who did not complete the intervention, all daily submission data up to their last submission of drink count or breath sample were included in the intention-to-treat analyses. All intention-to-treat analyses are included in the Supplemental Materials. For all statistical analyses, significance was set at p<.05. Follow-up pairwise comparisons for any significant main effects or interactions were conducted on estimated marginal means, and significance was based on Kenward-Rodger degrees of freedom^30^. All follow-up pairwise comparisons were corrected using the Tukey method.

Discounting data were screened for systematicity using the Johnson and Bickel criteria^31^; however, all data were analyzed, regardless of systematicity violations (**Tables S3–S4** show proportion of nonsystematic data). DD rates were calculated using Eq. 1^32^, where V represents the indifference point between the smaller-sooner and larger-later rewards, A represents the amount of the larger-later, *k* is the discounting rate, and D represents the delay. DD rates (*k*) were natural-logarithmic transformed (ln(*k*)) to fulfill the normality assumption. A linear mixed-effects model including fixed effects of group (EFT/CET), session (S1–S5), and group and session interaction, and a random intercept of participants were conducted using ln(*k*) as outcome. The model was adjusted for age, sex, education, income, and AUDIT score.


1 $$V=\frac{a}{1+kD}$$

Responses to the APT were screened for overconsumption, defined as purchases of more than 50 drinks/price for self-consumption over 24-hours. Thus, consumption during the purchase task was capped at 50 drinks at any given price, such that any number of drinks greater than 50 was transformed to 50 (n = 11 observations). Data from the purchase task were used to estimate intensity (i.e., consumption at price $0), alpha (i.e., elasticity of demand), O_max_ (i.e., maximum expenditure), and P_max_ (i.e., price at which O_max_ occurs) using the *beezdemand* package^33^. Alpha was estimated based on fits of Eq. 2^34^ to the data, where *Q* is the consumption at price *C*, *Q*_*0*_ estimates consumption at price $0, *α* is the elasticity of demand, and *k* is the span of the function.


2 $$Q=Q_{0}*10^{k(e^{-aQ_{0}C}-1)}$$

Separate generalized linear mixed-effects models including fixed effects of group (EFT/CET), session (S1–S5), and group and session interaction, and a random intercept of participants were conducted using each demand parameter (intensity, alpha, O_max_, and P_max_) as outcomes. Poisson distribution was used for the model on intensity. Alpha was natural-logarithmically transformed to fulfill the normality assumption. For the alpha analysis, data for which the equation did not converge were excluded (n = 2 in S1; n = 1 in S2; n = 2 in S3; n = 2 in S4; n = 2 in S5). All models were adjusted for age, sex, education, income, and AUDIT score.

Changes in alcohol consumption were analyzed using drinks/day and drinks/drinking day as outcomes. Separate generalized linear mixed-effects models including fixed effects of group (EFT/CET), phase (Baseline Monitoring, Monitoring Phase 1, and Monitoring Phase 2), and group and phase interaction, and a random intercept of participants were conducted for each outcome. Poisson distribution was used for both models. All models were adjusted for age, sex, education, income, and AUDIT score. For both the complete case and the intention-to-treat analyses, all missing drink counts were treated as missing.

Due to the high rate of missing breath sample data, the analysis on dichotomous breathalyzer results (positive or negative) described in the SAP was not performed. Instead, we performed a Fisher’s exact test to explore the association between the reported drinking days and the biochemically validated breath data. Additionally, we explored the area under the receiver operating characteristics (ROC) curve to determine the utility of peak breath sample (i.e., highest breath sample per day) to predict days in which a participant consumed at least one drink. For both the Fisher’s exact test and the ROC analysis, breath sample readings ≥ .02% of breath alcohol concentration were considered positive^25^. Lastly, we also explored the variables that impacted adherence to remote reporting (i.e., predicted missing daily samples) by performing an exhaustive search of the model space^35^. Separate model selections were performed using drink counts and breath samples submission statuses (i.e., submitted or missing) as outcomes. The predictors included in the model selection for each outcome were group, phase, group and phase interaction, and demographics of age, sex, ethnicity, race, education, and income. Additionally, the model selection for missing breath samples also included time of submission (i.e., first, second, or third submission of the day), and interactions between time of submission and phase, and time of submission and group as predictors. The optimal model was the one with the lowest Bayesian Information Criteria (BIC).

## Results

A total of 114 provided informed consent, 103 started the baseline monitoring phase, and 64 made it to the randomization phase and were randomized to EFT (n = 34) or CET (n = 30). Of those, 61 started the intervention (i.e., underwent the first cue generation; EFT = 33, CET = 28), and 52 completed the entire study, including the one-month follow-up (S6; EFT = 28, CET = 24). Reasons for discontinuation during intervention included voluntary withdrawal and loss to follow-up (i.e., participant stopped communication with the study team). Recruitment and data collection occurred between June 2022 and September 2024.

Table 1 summarizes baseline characteristics for EFT (n = 28) and CET (n = 24) participants who completed the entire study (**Table S5** includes baseline characteristics for all participants who started the intervention, n = 61). All participants met DSM-5 criteria for AUD. Groups were well balanced across demographic measures and randomization variables (i.e., drinks/day and ln(*k*)).

## Delay Discounting

Figure 2 shows ln(*k*) across sessions. The linear mixed-effects model for ln(*k*) indicated a significant Session and Group interaction (p<.001). The main effects of Session (*p*=.80) and Group (*p*=.45) were not significant (see **Table S6** for model estimates). Follow-up pairwise comparisons indicated that ln(k) was significantly lower in the EFT group during the cue generation sessions (S2 and S3) compared to both baseline (S1; *ps*<.001) and end-of-intervention (S4; *ps*<.01). No significant differences were observed between S2 and S3 (*p*=.99) or between S2/S3 and follow-up (S5; *ps*>.19). In contrast, ln(k) did not vary significantly across sessions in the CET group (*ps*>.86). Between-group differences were significant at S2 and S3 (*ps*<.02), but not at S1, S4, or S5 (*ps*>.41). These findings demonstrate that exposure to EFT, but not to CET, acutely reduced DD rates. Similar results were observed in the intention-to-treat analyses (see **Table S6**).

## Behavioral Economic Demand

The generalized mixed-effects models for intensity, alpha, O_max_, and P_max_ revealed no significant main effects of Group, Session, or Group and Session interaction. **Tables S7–S10** show the model estimates for each outcome. These results suggest that no reliable effects of EFT were observed on alcohol demand. Similar results were observed in the intention-to-treat analyses (see **Tables S7–S10**).

## Alcohol Consumption

**Figure 3:**
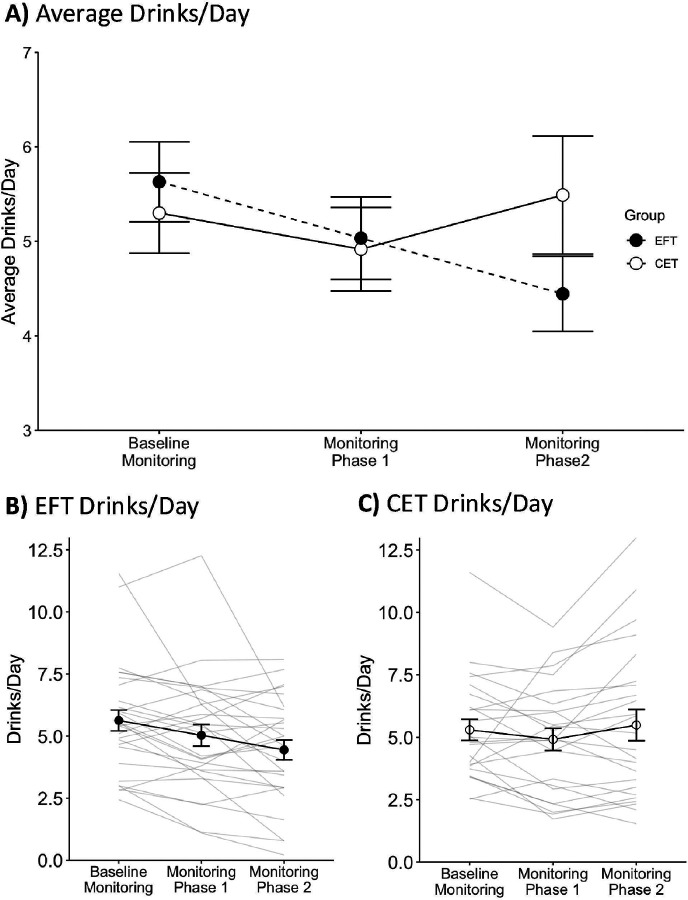
**A)** Average drinks/day across monitoring phases for EFT and CET. **B)** Drinks/day across monitoring phases for EFT. Grey lines represent individual data, and solid circles represent the group average. **C)** Drinks/day across monitoring phases for CET. Grey lines represent individual data, and open circles represent the group average. In all panels, error bars represent the standard error of the mean (SEM).

**Figure 4:**
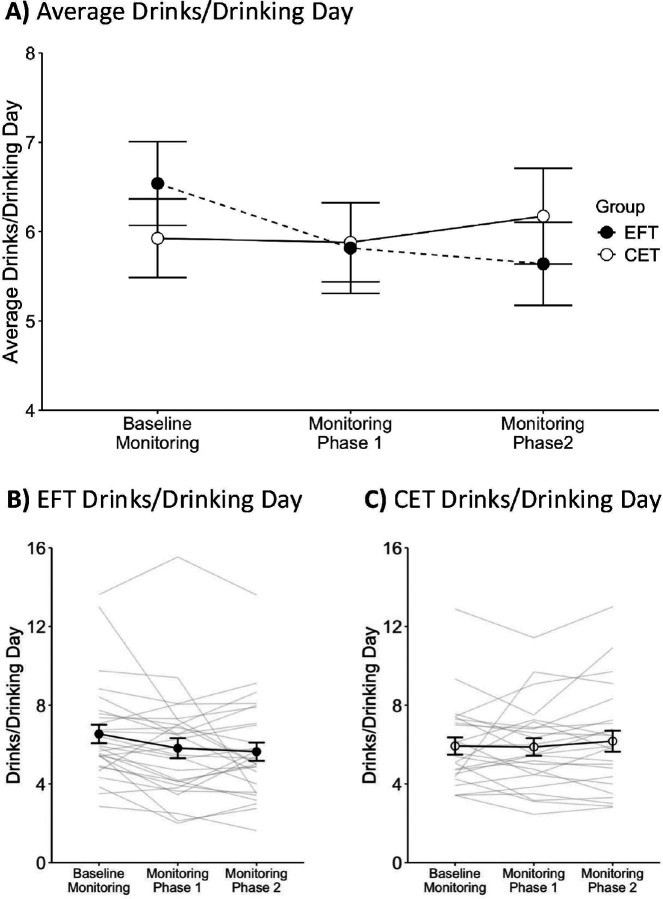
**A)** Average drinks/drinking day across monitoring phases for EFT and CET. **B)** Drinks/drinking day across monitoring phases for EFT. Grey lines represent individual data, and solid circles represent the group average. **C)** Drinks/drinking day across monitoring phases for CET. Grey lines represent individual data, and open circles represent the group average. In all panels, error bars represent the standard error of the mean (SEM).

Figures 3 and 4 show average drinks/day and average drinks/drinking day, respectively. Average drinks/day was calculated per participant by dividing the total drinks per phase by the total days in that phase. The average drinks/drinking day was calculated per participant by dividing the total drinks per phase by the number of days with at least one drink reported in that phase. The generalized mixed-effects model for drinks/day indicated a significant main effect of Phase (*p*>.01) and Phase and Group interaction (*p*<.001). The main effect of Group was not significant (*p*=.76; see **Table S11** for model estimates). Follow-up pairwise comparisons across phases indicated that the EFT group had a significantly lower drinks/day during the monitoring phase 1 (*p*<.01) and monitoring phase 2 (*p*<.001) relative to baseline monitoring, and during the monitoring phase 2 relative to the monitoring phase 1 (*p*<.001). For the CET group, drinks/day were significantly lower during the monitoring phase 1 relative to the baseline monitoring (*p*=.02) and to the monitoring phase 2 (*p*<.01) but not different between the monitoring phase 2 and baseline (*p*=.92). Pairwise comparisons between groups showed no significant differences in any phase (*ps*>.12).

The generalized mixed-effects model for drinks/drinking day indicated a significant Phase and Group interaction (*p*<.01). The main effects of Phase (*p*=.17) and Group (*p*=.60) were not significant (see **Table S12** for the model estimates). Follow-up pairwise comparisons across phases indicated that the EFT group had a significantly lower drinks/drinking day during the monitoring phase 1 (*p*<.01) and monitoring phase 2 (*p*<.01) relative to the baseline monitoring, but significant differences between the monitoring phases 1 and 2 were not observed (*p*=.77). For the CET group, pairwise comparisons did not indicate significant differences in drinks/drinking day across phases (*ps*>.14). Pairwise comparisons between groups showed no significant differences at any phase (*ps*>.28). Overall, these results suggest that drinks/day and drinks/drinking day consistently decreased across phases for the EFT but not for the CET group.

We observed a higher proportion of missing breath samples than missing drink counts throughout the study (see **Table S13** for proportion of submitted drink counts and breath samples). Despite the high proportion of missing breath samples, the Fisher’s exact test showed a significant association (OR: 16.60; *p*<.001) between breath sample results (positive or negative sample) and drink counts (positive or negative drink count), where the odds of a positive breath sample was about 16 times higher when participants reported at least one drink compared to zero drinks. Additionally, the AUC for the ROC curve was 0.79, indicating moderate discrimination. Table 2 shows the matrix comparing breath sample and drink count results. Noteworthy, most inconsistent responses correspond to individuals who had negative breath samples but self-reported at least one drink. One possible reason for this inconsistency is that participants could have provided a negative breath sample before starting drinking and not provided the following samples for that day. Notably, all findings for the alcohol consumption were replicated in the intention-to-treat analyses (see **Tables S11-S14**).

Results from the model selection indicated that phase was the best predictor for missing drink count and missing breath samples. For both outcomes (i.e., drink count and breath samples), the odds of missing a daily submission during the monitoring phase 2 were significantly higher than for the other two monitoring phases, but not different between baseline and monitoring phase 1 (see **Tables S15-S16** for model estimates). Notably, group assignment was not a predictor of missing drink count and breath samples. These results suggest that compliance with the remote procedures decreased with time into the study, regardless of group assignment. These findings were replicated in the intention-to-treat analyses (see **Tables S15-S16**).

## Discussion

The present study evaluated the effects of EFT on DD, behavioral economic demand, and real-world alcohol consumption in a non-treatment-seeking AUD sample. Aligned with our hypotheses, the results showed 1) decreases in DD rates in the presence of the EFT, but not CET, cues, and 2) decreases in drinks/day and drinks/drinking day with daily exposure to EFT, but not to CET. However, contrary to our hypothesis, EFT did not impact alcohol demand.

First, the present results replicate previous findings showing that EFT decreases DD rates^8,36^. Notably, in the present study, EFT only decreased DD rates when the cues were presented during the task. Thus, although EFT increases preference for the delayed reinforcers, those effects might not generalize when the cues are not available. Studies exploring how to increase the generalizability and duration of EFT effects are warranted before we can fully understand EFT’s long-term effects on decision-making.

Second, the present results replicate and extend previous findings^16,37^ showing that EFT decreases real-world alcohol consumption (measured as either drinks/day or drinks/drinking day). Furthermore, we showed this reduction across a 4-week intervention following two cue generation sessions, an extension of the previous study that investigated changes in drinking during a 2-week intervention following one cue generation. Thus, the present results demonstrate that daily exposure to EFT can sustain reduced alcohol consumption for a longer period than previously demonstrated. However, significant differences between groups were not observed. The lack of significant group effects in daily drinking remains unclear. Notably, EFT decreased alcohol consumption in individuals with AUD who had a desire to quit but no immediate plans to enroll in treatment. Thus, EFT might be efficacious for non-treatment-seeking individuals.

Lastly, we did not observe EFT effects on alcohol demand. The reason for the lack of effect is unclear, especially considering the observed changes in alcohol consumption. One possible explanation for this lack of effect could be an insensitivity of the task to capture changes in valuation. For example, although the instructions specified the standard sizes of different types of drinks (e.g., 12 oz beer, 5 oz glass of wine, 1.5 oz shot of hard liquor), we did not ask what type of alcoholic beverage participants were considering when making their choices during the task. EFT might have impacted the type of drink purchased, and in turn the unit price (e.g., price per ounce) the participant was working with across sessions. Thus, purchase patterns may have changed across sessions in ways that were not captured by number of drinks purchased. Additionally, during the task, participants were instructed to purchase for 24-hour consumption. Thus, participants could have responded in a way that was representative of their typical drinking pattern instead of the pattern they intended to follow moving forward. It is also important to note that EFT effects on demand parameters have not been consistently observed^38^ and are mainly observed for intensity^22,39^. Thus, more studies investigating the optimal conditions under which EFT impacts demand parameters are needed.

Nonetheless, significant decreases in daily alcohol consumption were observed, even in the absence of changes in demand. This finding may indicate that EFT was effective in decreasing alcohol valuation, without impacting demand or that our assessment of demand needs further refinement (including possibly real-world purchase behavior). Alternatively, this finding may suggest that, despite the theoretical prediction, extending the temporal window may reduce consumption independent of changing alcohol reinforcing value. In that case, EFT might affect decision-making pathways other than reinforcement value. For example, EFT may impact motivation to consume without necessarily decreasing the value associated with the reinforcer.

Some limitations are worth noting. First, most of the sample was white, educated, and currently employed, which might limit the generalizability of the results. Second, we observed an increasing proportion of missing data for the daily reports with study progression, which might indicate that the remote monitoring was burdensome and led to decreased compliance with extended participation. This might have been especially true for participants who had to use a study phone, instead of their own phones, for their daily submissions. Additionally, the time windows for breath sample submissions were random and could happen at inconvenient times for the participants. Similarly, the daily cue reminders were sent at fixed times, which could not align with peak craving or drinking time. Thus, whether more robust effects could have been observed with more frequent reminders or self-selected time windows remains unclear. Third, the length of the monitoring phases was contingent on session completion; thus, participant and staff availability for the sessions impacted phase duration. This difference in phase length should not impact the conclusions about changes in consumption, given that consumption analyses accounted for the number of days in each phase. However, it is unclear whether the additional EFT exposure for those with longer phases could have a differential impact on the outcomes. Fourth, our measure of drinks/day was based on self-report. Although we showed a significant association between breath sample results and drink counts, providing biochemical verification of our self-reported data, we recognize that this association does not provide evidence of the accuracy of the number of drinks reported. Thus, direct measures of drinking behavior are recommended in future trials. Additionally, linking the cue exposure more directly to observed drinking behavior could produce more robust effects of EFT on drinking.

Adherence to the intervention could be improved in future studies by using patient-centered approaches in study design, allowing participants to select most convenient/effective monitoring and cue reminder windows, or tailoring these windows based on drinking patterns or ecological momentary assessments of craving. The use of less burdensome biological verification of alcohol use, such as transdermal alcohol sensors, could also decrease barriers to compliance and improve adherence to the intervention. Furthermore, studies are still needed to identify the optimal conditions under which EFT produces generalizable and long-lasting effects. Studies including longer follow-up monitoring and drinking measures post-intervention are also needed to evaluate whether the decrease in drinking is sustained after discontinuation of the daily reminders. Future studies should also include a more diverse population, especially underserved populations that are more largely affected by AUD. This could be achieved, for example, by using targeted recruitment strategies and including individuals with lived experiences in the recruitment and study design team.

Overall, the present findings provide evidence of EFT effectiveness in decreasing discounting and real-world daily drinking. Notably, daily exposure to EFT decreased alcohol consumption in individuals with AUD who had a desire to quit but no immediate plans to enroll in treatment. EFT may be a particularly relevant intervention given its easy implementation with remote delivery, allowing it to be used with hard-to-reach populations^24^ and integrated with other therapeutic approaches to augment their effect^40,41^. For example, EFT may be useful as an adjunctive therapy to other digital approaches such as app-based contingency management or cognitive behavioral therapy. EFT may be especially complementary to contingency management approaches, as contingency management is sometimes associated with short-term effects, while the decrease in discounting associated with EFT may lead to longer-term effects.

Furthermore, EFT might be more acceptable for non-treatment-seeking individuals, as it does not focus on alcohol specifically. Future studies can continue to inform how EFT can be best tailored to produce the most effective and robust results.

## Figures and Tables

**Table 1:** Sample demographics

n	EFT	CET
	24	28
Age^a^	37.21 (12.59)	41.32 (13.39)
Sex = Male^b^	16 (66.7)	17 (60.7)
Ethnicity = NOT Hispanic or Latino^b^	23 (95.8)	26 (92.9)
Race^b^		
Asian	1 (4.2)	0 (0.0)
Black or African American	3 (12.5)	4 (14.3)
More Than One Race	1 (4.2)	1 (3.6)
Other	1 (4.2)	0 (0.0)
White	18 (75.0)	23 (82.1)
Education^b^		
High School (9 + years)	5 (20.8)	5 (17.9)
College (13 + years)	15 (62.5)	17 (60.7)
Graduate School (17 + years)	4 (16.7)	6 (21.4)
Employment^b^		
Retired	3 (13.0)	1 (3.6)
Unemployed	2 (8.7)	1 (3.6)
Working full time	12 (52.2)	19 (67.9)
Working part-time	6 (26.1)	7 (25.0)
Income^b^		
Less than $5,000	2 (8.3)	4 (14.3)
$5,000 through $24,999	4 (16.7)	5 (17.9)
$25,000 through $49,999	8 (33.3)	7 (25.0)
$50,000 through $74,999	3 (12.5)	4 (14.3)
$75,000 through $99,999	2 (8.3)	4 (14.3)
$100,000 and greater	4 (16.7)	4 (14.3)
No response	1 (4.2)	0 (0.0)
Baseline DD rate (ln(k))^a^	−5.09 (2.54)	−4.83 (2.53)
AUDIT^a^	21.92 (3.68)	24.68 (4.90)
AUD score^a^	8.42 (2.02)	7.75 (2.55)
CannaNs Use Disorder	1.08 (1.82)	0.93 (2.11)
To3acco Use Disorder	0.04 (0.20)	0.00 (0.00)
Opioid Use Disorder (Mild)	0.04 (0.20)	0.00 (0.00)
Stimulants Use Disorder (Mild)	0.96 (1.99)	1.28 (3.02)
Cocaine Use Disorder (Mild)	0.00 (0.00)	0.11 (0.57)
Baseline drinks/day^a^	5.63 (2.23)	5.30 (2.08)

Note:

a Mean (SD);

b Count (%)

**Table 2:** Matrix of breath samples and self-reported drink counts.

		Self-Reported Drinks		
Breath Samples		None	One or more	Missing
	Negative	184	519	103
	Positive	18	844	75
	Missing	108	426	242
